# FLAIR-only joint volumetric analysis of brain lesions and atrophy in clinically isolated syndrome (CIS) suggestive of multiple sclerosis

**DOI:** 10.1016/j.nicl.2020.102542

**Published:** 2020-12-25

**Authors:** O. Goodkin, F. Prados, S.B. Vos, H. Pemberton, S. Collorone, M.H.J. Hagens, M.J. Cardoso, T.A. Yousry, J.S. Thornton, C.H. Sudre, F. Barkhof, MAGNIMS study group

**Affiliations:** a Centre for Medical Image Computing (CMIC), University College London, London, United Kingdom; b Neuroradiological Academic Unit, UCL Queen Square Institute of Neurology, University College London, London, United Kingdom; c eHealth Centre, Universitat Oberta de Catalunya, Barcelona, Spain; d Lysholm Department of Neuroradiology, National Hospital for Neurology and Neurosurgery, UCLH NHS Foundation Trust, London, United Kingdom; e NMR Research Unit, Queen Square Multiple Sclerosis Centre, Department of Neuroinflammation, UCL Institute of Neurology, Faculty of Brain Sciences, University College London (UCL), London, United Kingdom; f MS Center Amsterdam, Department of Neurology, Amsterdam UMC, Vrije Universiteit Amsterdam, Amsterdam, The Netherlands; g School of Biomedical Engineering and Imaging Sciences, King’s College London, London, United Kingdom; h Dementia Research Centre, UCL Queen Square Institute of Neurology, University College London, London, United Kingdom; i Radiology & Nuclear Medicine, VU University Medical Center, Amsterdam, Netherlands

**Keywords:** Magnetic resonance imaging, Multiple sclerosis, Demyelinating diseases, Neurodegenerative diseases

## Abstract

**Background:**

MRI assessment in multiple sclerosis (MS) focuses on the presence of typical white matter (WM) lesions. Neurodegeneration characterised by brain atrophy is recognised in the research field as an important prognostic factor. It is not routinely reported clinically, in part due to difficulty in achieving reproducible measurements. Automated MRI quantification of WM lesions and brain volume could provide important clinical monitoring data. In general, lesion quantification relies on both T1 and FLAIR input images, while tissue volumetry relies on T1. However, T1-weighted scans are not routinely included in the clinical MS protocol, limiting the utility of automated quantification.

**Objectives:**

We address an aspect of this important translational challenge by assessing the performance of FLAIR-only lesion and brain segmentation, against a conventional approach requiring multi-contrast acquisition. We explore whether FLAIR-only grey matter (GM) segmentation yields more variability in performance compared with two-channel segmentation; whether this is related to field strength; and whether the results meet a level of clinical acceptability demonstrated by the ability to reproduce established biological associations.

**Methods:**

We used a multicentre dataset of subjects with a CIS suggestive of MS scanned at 1.5T and 3T in the same week. WM lesions were manually segmented by two raters, ‘manual 1′ guided by consensus reading of CIS-specific lesions and ‘manual 2′ by any WM hyperintensity. An existing brain segmentation method was adapted for FLAIR-only input. Automated segmentation of WM hyperintensity and brain volumes were performed with conventional (T1/T1 + FLAIR) and FLAIR-only methods.

**Results:**

WM lesion volumes were comparable at 1.5T between ‘manual 2′ and FLAIR-only methods and at 3T between ‘manual 2′, T1 + FLAIR and FLAIR-only methods. For cortical GM volume, linear regression measures between conventional and FLAIR-only segmentation were high (1.5T: α = 1.029, R^2^ = 0.997, standard error (SE) = 0.007; 3T: α = 1.019, R^2^ = 0.998, SE = 0.006). Age-associated change in cortical GM volume was a significant covariate in both T1 (p = 0.001) and FLAIR-only (p = 0.005) methods, confirming the expected relationship between age and GM volume for FLAIR-only segmentations.

**Conclusions:**

FLAIR-only automated segmentation of WM lesions and brain volumes were consistent with results obtained through conventional methods and had the ability to demonstrate biological effects in our study population. Imaging protocol harmonisation and validation with other MS phenotypes could facilitate the integration of automated WM lesion volume and brain atrophy analysis as clinical tools in radiological MS reporting.

## 1 Introduction

Magnetic resonance imaging (MRI) assessment is fundamental for diagnosis and monitoring in multiple sclerosis (MS). MS is a demyelinating disease of the central nervous system characterised by inflammation and neurodegeneration (Sand, 2015). A patient’s initial symptomatic demyelinating event is referred to as clinically isolated syndrome (CIS), and where brain MRI lesions have a pattern consistent with MS, these patients have a high probability of converting to relapsing-remitting MS in the future (Kappos et al., 2007). Radiological evaluation focuses on the presence of MS-typical white matter lesions, in terms of their morphology and location. Once MS has become established, change in lesion load over time and in response to treatment is the focus of radiology reporting. Another component of MS pathology – namely neurodegeneration characterised by brain atrophy - has been recognised as an important prognostic factor for disease progression in the research field (Sastre-Garriga et al., 2017). It is not routinely reported in the clinical setting and not included in diagnostic or monitoring guidelines (Thompson et al., 2018, Lublin et al., 2014), in part because of difficulty in achieving reproducible measurements (Sastre-Garriga et al., 2020).

The interpretation provided by the radiologist could benefit from embedding automated volumetric lesion and brain volume assessments into the clinical routine setting. Efforts have recently been made towards clinically useful solutions that take into account image quality and acquisition heterogeneity that is common in clinical settings (Zivadinov et al., 2018, Dwyer et al., 2019), by using T2 weighted-Fluid Attenuation Inversion recovery, T2-FLAIR, to not only measure lesion volume but also determine central atrophy in a reproducible fashion using heterogeneous clinical data.

Volumetric techniques for total lesion load and brain volume quantification have been developed in the research and clinical trial settings, where image acquisition is more homogeneous and multiple contrasts are available (Lindig et al., 2018, Danelakis et al., 2018). In general, lesion segmentation techniques rely on the availability of multi-contrast source image data sets, i.e. requiring both T1- and T2-weighted (e.g. T2-FLAIR) images, with automated techniques typically reliant on isotropic three dimensional (3D) acquisitions but manual delineations often performed on two dimensional (2D) acquisitions (Simões et al., 2013, de Boer et al., 2009). Brain volume quantification solutions typically require a 3D T1-weighted image dataset. Segmentation accuracy is affected by the presence of white matter lesions and can be improved by detecting and correcting for them (Valverde et al., 2015).

In the routine work-up of MS patients, a 3D T1-weighted scan is generally not part of the clinical MRI protocol (Schmierer et al., 2019). While there are several proprietary solutions available for lesion segmentation and brain volume quantification, these require 3D T1-weighted, as well as T2-FLAIR images, and are variable in the information they offer, some providing only lesion segmentation or brain volumetry (Jain et al., 2015). Moreover, it is difficult to gauge how these solutions have been validated and what gold standard they have been assessed against (Wilkinson and van Boxtel, 2019). All these problems present a substantial barrier for translation of valuable quantitative techniques for well-validated implementation for clinical radiological use in MS.

In this study, we aim to address an aspect of this important translational challenge, namely that of non-standard sequence availability, which is one amongst the many required to achieve clinical implementation of an automated imaging biomarker tool (Goodkin et al., 2019). We will do this by assessing the performance of T2-FLAIR-only simultaneous lesion segmentation and brain volume quantification and comparing against a conventional approach for lesion and brain tissue segmentation requiring a multi-contrast acquisition, namely T1 and T2-FLAIR. We will investigate whether the output from an automated lesion segmentation tool is more reflective of manual segmentation of all white matter hyperintensities (WMH) or only typical MS lesions. We will explore the reproducibility of imaging biomarker extraction by applying the methods to a multi-centre, multi-vendor dataset of subjects with a CIS suggestive of MS scanned in both 1.5T and 3T scanners within the same week (Hagens et al., 2018), which will allow us to evaluate the performance of automated lesion and brain segmentation at the two field strengths.

We aim to establish the extent to which T2-FLAIR-only lesion and brain segmentation introduces more variability in performance compared with conventional segmentation. We will explore the effects of field strength and WM lesion inpainting (Chard et al., 2010, Prados et al., 2016); and whether the results reflect established biological associations, for example age-related changes in brain volume. We hypothesise that T2-FLAIR-only segmentation will achieve comparable results to conventional methods.

## 2 Methods

### 2.1 Dataset

We used the dataset described by Hagens et al. (2018), which consists of CIS subjects recruited between July 2013 and September 2015 from six European MS centres in the Magnetic Resonance Imaging in Multiple Sclerosis (MAGNIMS) network (www.magnims.eu). For the purposes of this study we used a subset of 66 CIS subjects.

Inclusion criteria for CIS subjects were defined by the international panel on MS diagnosis (Polman et al., 2011), and all subjects included were aged between 18 and 59 years at baseline, with no other immunological, vascular or oncological medical history. Local institutional review boards approved the study at each centre and all participants gave their written informed consent to participate.

### 2.2 MRI acquisition

MRI was performed at both 1.5T and 3T, within the same week. Scanning parameters were applied in accordance with the MAGNIMS guidelines (Wattjes et al., 2015) using a multisequence scanner optimised acquisition protocol (Hagens et al., 2018). In particular, acquisitions included isotropic gradient echo 3-D T1-weighted (T1) and 3D turbo spin echo T2-FLAIR. Acquisition parameters for each centre can be found in the supplementary material.

### 2.3 WM lesion detection

Consensus joint reading was performed for all scans using a digital workstation (Sectra [Linköping, Sweden] IDS7 version 16.2.28) by three experienced readers in random order, with a minimum reading time interval of two weeks between 1.5T and 3T scans, as described (Hagens et al., 2018). Lesions were defined as all areas of abnormal white matter hyperintensity consistent with CIS apparent on T2-FLAIR images and larger than 3 mm diameter. The raters had knowledge of the localisation of initial symptoms and signs detected by the neurologist but they were not informed of subject age, gender or centre.

### 2.4 Manual WM lesion segmentation

In order to assess whether automated lesion segmentation resembles segmentation of any WMH or typical MS lesions, we performed two types of manual segmentation. Rater 1 (OG) performed manual segmentation of baseline lesions using NiftyMIDAS (Clarkson et al., 2015) guided by the expert consensus labelling described in 2.3, referred to in results as manual method 1. Rater 2 (SC) performed separate manual segmentation in 3D slicer (Pieper et al., 2004), a comparable toolkit (Gibson et al., 2018), on a subset of subjects, not guided by the expert consensus lesion labelling, to include any hyperintensity, referred to in results as manual method 2.

### 2.5 Automated WM lesion segmentation

Two sequence input segmentation was performed on baseline T1 and T2-FLAIR images using the Bayesian Method of Model Selection (BaMoS) (Sudre et al., 2015). Briefly, this is an unsupervised hierarchical model selection framework which enables the distinction between different types of expected and abnormal signal intensities within the white matter (after brain parcellation, see below). Single sequence lesion segmentation was repeated on the same dataset using BaMoS with the T2-FLAIR as the only input sequence. Similarly to the original method using jointly T1 and T2-FLAIR, a Gaussian mixture model was fitted to the data, optimising the number of components required for each tissue class and using the output of the parcellation obtained using a database uniquely composed of T2-FLAIR images to perform the post-processing dedicated to removal of false positives.

### 2.6 Brain tissue segmentation

Brain tissue segmentation was performed using a fully automated multi-atlas-based approach, Geodesic Information Flows (GIF), (Cardoso et al., 2015).

This was done using 1) a 3D T1 image database (the original GIF database composed of images manually labelled by expert operators (Cardoso et al., 2015); or 2) a newly-constructed GIF database, containing both 3D T1 and 3D T2-FLAIR images. This new database was constructed using 100 healthy control subjects’ (age range 46–90 years, mean age 72, 51.1% males) coregistered 3D T1 and 3D T2-FLAIR images from the SABRE study cohort (Tillin et al., 2012) with the following acquisition parameters: 3D sagittal T1 multishot, inversion-prepared gradient echo: repetition time 6.9 ms; echo time 3.1 ms; voxel size 1.0×1.0×1.0 mm^3^; and 3D sagittal T2-FLAIR: repetition time 4800 ms; inversion time 1650 ms; echo time 125 ms; voxel size 1.0×1.0×1.0 mm^3^. The new T1 images were automatically segmented using the original T1 labels which were then propagated to the T2-FLAIR images. The performance of the GIF algorithm with the original and new GIF databases were compared conventionally by segmenting the CIS cohort’s 3D T1 images for direct comparison of the effect of database change. GIF segmentation using the combined database was then tested with 3D T1 only, and T2-FLAIR only as the source images. In order to assess the performance of tissue segmentation in those subjects with high white matter lesion loads, we performed a subset analysis of the 10% of cases with the largest lesion volumes. T2-FLAIR images were registered to T1 space before segmentation to allow for voxel-wise comparisons. Performance using these image inputs was tested with varying degrees of WM lesion inpainting (Chard et al., 2010) using a patch-based method (Prados et al., 2016): 1) uncorrected, 2) manual WM lesion filled and 3) BaMoS outlier filled.

### 2.7 Statistical analysis

#### 2.7.1 WM lesions

We assessed 1) median and interquartile range (IQR) of absolute lesion volume, and 2) percentage lesion volume difference, by segmentation method and field strength. We also compared differences with related-samples Wilcoxon signed rank tests. We used the Dice similarity coefficient (DSC) to compare similarity between the reference (conventional multiple sequence input) and T2-FLAIR-only sample. DSC is calculated as:

$$\text{DSC}=\frac{2\text{TP}}{2\text{TP}+\text{FP}+\text{FN}}$$

Where TP = true positive, FP = false positive, and FN = false negative.

Proportion of lesion volume difference between conventional and T2-FLAIR-only BaMoS methods was calculated as (T2-FLAIR-only volume – conventional volume / conventional volume). Median percentage volume difference was calculated as median (conventional volume – T2-FLAIR-only volume / average volume)*100.

#### 2.7.2 Brain volumetry

We used paired t-tests to compare brain volume group means between T1 and T2-FLAIR GIF. We compared brain volume results of tissue classes (GM, WM and CSF) between T1 and T2-FLAIR inputs into the GIF database using a no-intercept linear regression. Linear regression modelling was performed for 3 main tissue classes – cerebrospinal fluid (CSF), WM, and GM – and the combined total intracranial volume (TIV) for the same segmentation method comparisons. A no-intercept model was used in line with the expected unity between methods. Calculations were made for model fit (Akaike Information Criterion, AIC) for both intercept and no-intercept models. We also performed a subset analysis for the 10% of subjects with the highest WM lesion loads, to assess tissue segmentation performance in more radiologically advanced disease.

The clinical utility of T2-FLAIR-only volumetry was assessed by evaluating the ability to demonstrate age differences. Since we used a CIS cohort with little disease-related atrophy developed, we used a general linear model to assess brain volume effects of age for both methods. We calculated effect sizes (Cohen’s f, 2013), where values 0.10, 0.25 and 0.40 represent small, medium and large effect sizes respectively,) to demonstrate the number of cases that would be needed to show group differences for age using the adapted methods. Statistical analysis was performed using SPSS for Windows, Version 25.0. Armonk, NY: IBM Corp.

## 3 Results

66 patients with CIS were included in this study. Their mean age was 34.7 years (±8.4), and 47 were female, with a median Expanded Disability Status Scale (EDSS) score of 2.0 (range 0–6.0).

### 3.1 Manual and automated assessment of WMH and MS lesions

**Fig. 1:**
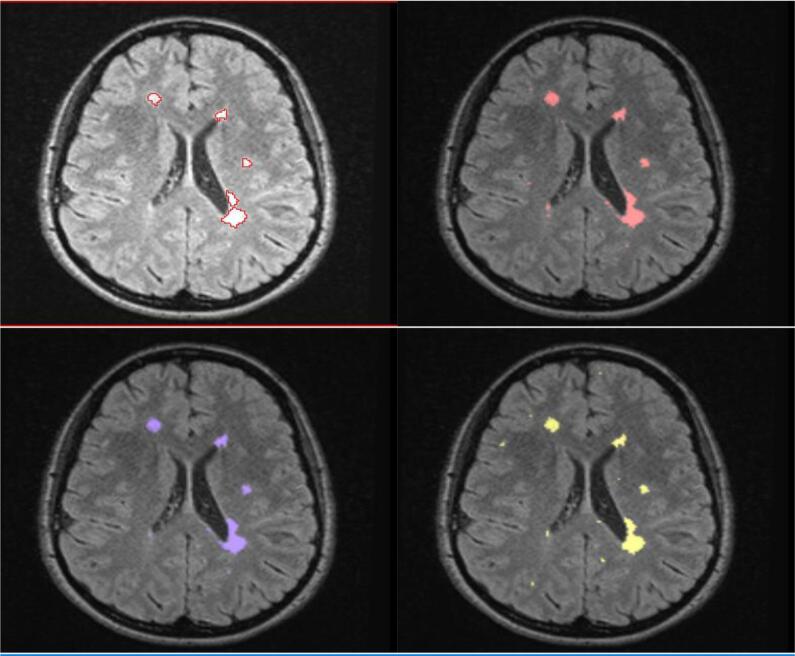
An example of WM lesion segmentation results for manual method 1, top left; manual method 2, top right; multi-sequence BaMoS, bottom left; and FLAIR-only BaMoS, bottom right.

**Fig. 2:**
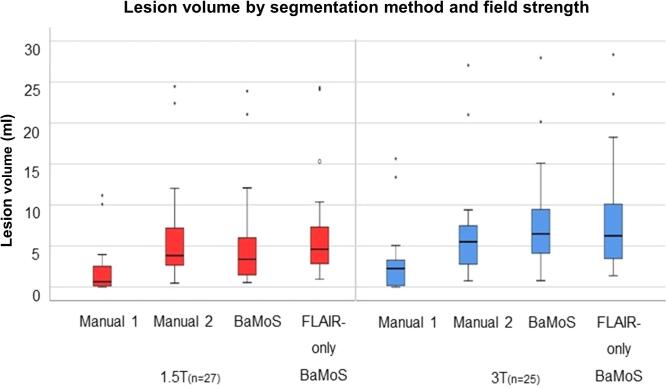
Boxplots showing median, IQR and range for lesion volume in mm3 per segmentation method by field strength.

Wilcoxon signed rank tests comparing total lesion volume between methods showed statistically significant differences between manual segmentation method 1 and all other methods, with method 1 producing lower lesion volumes at both 1.5T and 3T, p < 0.001. For 1.5T, lesion volumes segmented with manual method 2 were not significantly different to T2-FLAIR-only BaMoS (p = 0.239). Conventional (T1 + T2-FLAIR) and T2-FLAIR-only BaMoS produced significantly different lesion volumes at 1.5T (p = 0.01), with T2-FLAIR-only BaMoS producing larger lesion volumes. At 3T however, manual method 2 was not significantly different to conventional BaMoS (p = 0.231) as were conventional and T2-FLAIR-only BaMoS methods, p = 0.819. Median lesion volume in ml (IQR) by segmentation method is shown in Table 1 and graphically represented in Fig. 2. An example of segmentations obtained using the four methods of WM lesion segmentation for one subject is shown in Fig. 1.

**Table 1:** Median lesion volume and interquartile range (IQR) for each segmentation method and field strength.

Lesion segmentation method	Field strength	Median lesion volume (ml)	Inter-quartile range (IQR)
Manual 1	1.5T	0.63	2.44
	3T	2.25	3.17
Manual 2	1.5T	3.84	4.83
	3T	5.51	4.88
BaMoS	1.5T	3.38	5.03
	3T	6.48	5.90
T2-FLAIR-only BaMoS	1.5T	4.61	4.81
	3T	6.25	6.95

**Fig. 3:**
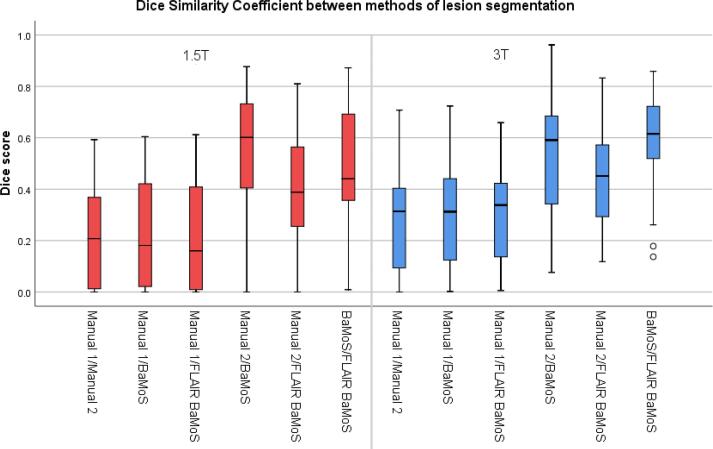
Boxplots representing dice similarity coefficient values between methods by field strength.

Mean DSC (SD) between conventional BaMoS and T2-FLAIR-only BaMoS are 0.46 (0.24) for 1.5T and 0.57 (0.19) for 3T (Fig. 3). Dice similarity coefficients (DSC) between lesion segmentation methods are shown in Table 2.

**Fig. 4:**
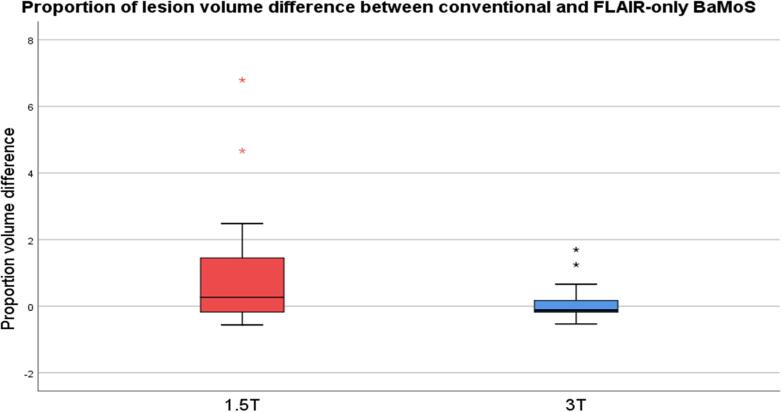
Proportion of volume difference between conventional and T2-FLAIR-only BaMoS lesion segmentation at 1.5T and 3T.

**Table 2:** Dice similarity coefficients between lesion segmentation methods by field strength. SD, standard deviation.

Lesion segmentation method comparison	Field strength	Dice similarity coefficient Mean (SD)
Manual 1 vs Manual 2	1.5T	0.21 (0.20)
	3T	0.28 (0.21)
Manual 1 vs BaMoS	1.5T	0.25 (0.23)
	3T	0.32 (0.22)
Manual 2 vs BaMoS	1.5T	0.52 (0.25)
	3T	0.53 (0.24)
Manual 1 vs T2-FLAIR-only BaMoS	1.5T	0.21 (0.21)
	3T	0.29 (0.20)
Manual 2 vs T2-FLAIR-only BaMoS	1.5T	0.37 (0.23)
	3T	0.43 (0.19)
BaMoS vs T2-FLAIR-only BaMoS	1.5T	0.46 (0.24)
	3T	0.57 (0.19)

Proportion of lesion volume difference between conventional and T2-FLAIR-only BaMoS methods was median (IQR) 0.33 (−1.75 – 1.45) for 1.5T, and −0.13 (−1.87 – 0.18) for 3T (Fig. 4). Median percentage volume difference was −28.7% for 1.5T and 13.6% for 3T.

### 3.2 Brain tissue volumes

**Fig. 5:**
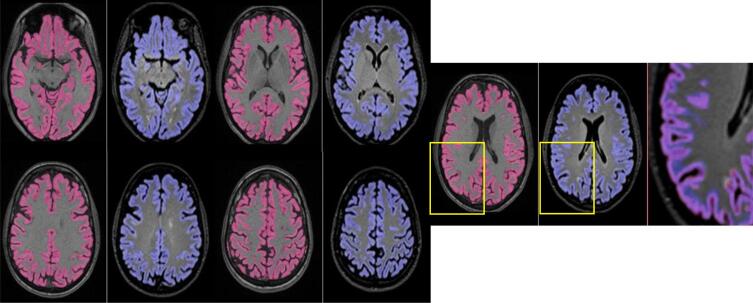
A subject’s cortical GM segmentation shown for 1.5T, using the multimodal GIF database. T1 segmentation is denoted in pink, and T2-FLAIR segmentation is shown in blue. An enlarged image overlaying both T2-FLAIR and T1 segmentations is included on the right of each series, showing areas of discrepancy, highlighted in the yellow boxes.

**Fig. 6:**
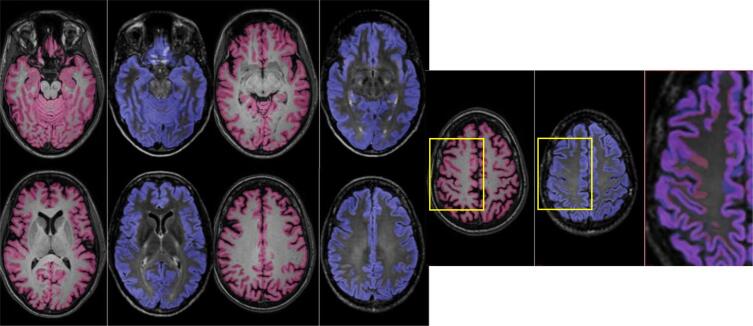
A subject’s cortical GM segmentation shown for 3T, using the multimodal GIF database. T1 segmentation is denoted in pink, and T2-FLAIR segmentation is shown in blue. An enlarged image overlaying both T2-FLAIR and T1 segmentations is included on the right of each series, showing areas of discrepancy, highlighted in the yellow boxes.

Mean cortical grey matter volume for each of three key segmentation methods are presented in Table 3 according to field strength. These results are for 1) original GIF database with T1 input, where GM volume (ml) was mean (SD) 503.4 (5.93) at 1.5T and 501.8 (6.10) at 3T, and for multi-modal GIF database with 2) T1 input (515.5 (6.04) at 1.5T and 512.7 (6.12) at 3T) and 3) T2-FLAIR input (529.8 (7.30) at 1.5T and 523.0 (6.77) at 3T. WM lesion inpainting results are shown in supplementary material and did not significantly alter GM volume measurements. All results presented in the main text have been processed with WM lesion inpainting using results from BaMoS WM segmentation. All three combinations of paired samples t-tests performed separately for 1.5T and 3T showed significant differences, at p < 0.001, with higher mean GM values produced by T2-FLAIR input at both 1.5T and 3T. Examples of GM segmentation results are shown in Figs. 5 and 6.

**Table 3:** GM volume in ml by GIF method (input and database). Mean volume, standard deviation (SD).

Descriptive statistics		Field strength	Mean GM Volume (ml)	SD
Input	GIF Database			
T1	original	1.5T	503.4	5.93
T1	original	3T	501.8	6.10
T1	new	1.5T	515.5	6.04
T1	new	3T	512.7	6.12
T2-FLAIR	new	1.5T	529.8	7.30
T2-FLAIR	new	3T	523.0	6.77

**Fig. 7:**
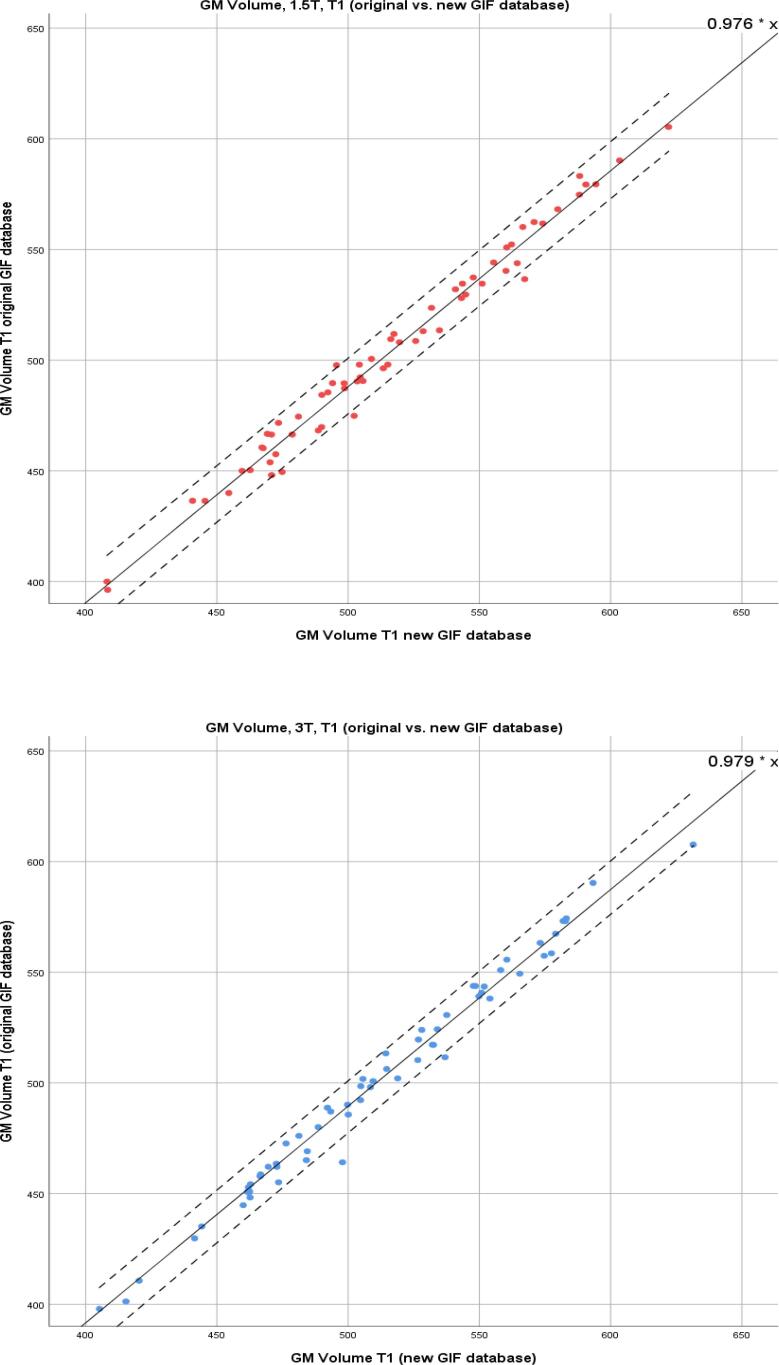
Scatter plots for GM volumes in ml; T1 input into conventional and new GIF database. Left: 1.5T. Right: 3T. Coefficient shown in upper right-hand corner and 95% CI shown with dotted lines.

**Fig. 8:**
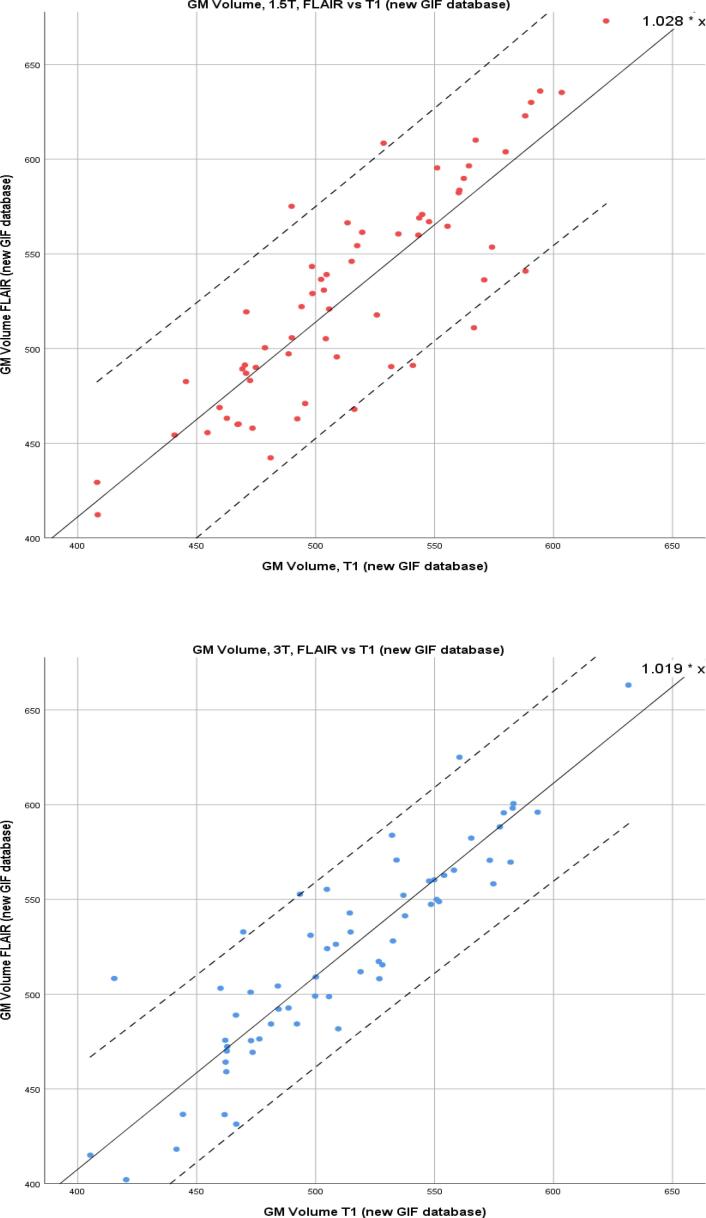
Scatter plots for GM volumes in ml; T2-FLAIR vs. T1 input into new GIF database. Left: 1.5T. Right: 3T. Coefficient shown in upper right-hand corner and 95% CI shown with dotted lines.

Linear regression modelling was performed for CSF, WM, and GM and the combined total intracranial volume (TIV) for the same segmentation method comparisons. AIC calculations showed no evidence of model fit deterioration (see supplementary material). The results for T1 and T2-FLAIR (using the new GIF database), demonstrating the effect of changing the inputted sequence, are shown in Table 4. For GM volume at 1.5T R^2^ was 0.997, β (SE) 1.028 (0.007), and at 3T R^2^ was 0.998, β (SE) 1.019 (0.006). For model results where there is a change of GIF database see supplementary material. GM correlations are illustrated in Fig. 7, Fig. 8, demonstrating the important comparisons – change of GIF database, and change of input sequence - by field strength. They show that there is a widening of the 95% confidence intervals for the correlation between T1 and T2-FLAIR GM volumes.

**Table 4:** Linear regression outputs for comparison of T1 and T2-FLAIR inputs into the new GIF database.

Change of sequence input			
-T1 vs T2-FLAIR input		β (SE)	R^2^
GM	1.5T	1.028 (0.007)	0.997
	3T	1.019 (0.006)	0.998
WM	1.5T	0.995 (0.007)	0.997
	3T	1.055 (0.008)	0.996
CSF	1.5T	0.944 (0.012)	0.989
	3T	0.859 (0.009)	0.994
TIV	1.5T	0.973 (0.004)	0.999
	3T	0.999 (0.004)	0.999

β = slope coefficient, SE = standard error.

**Fig. 9:**
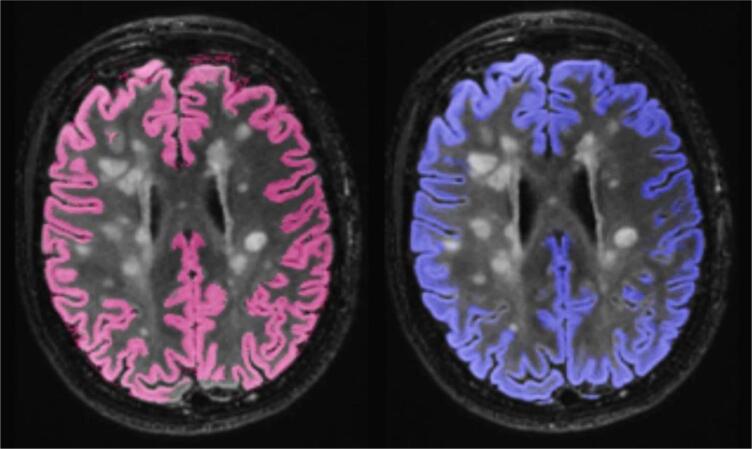
GM segmentation performance in the context of high WM lesion load, using the new GIF database (pink = T1, blue = FLAIR).

To address generalisability of our findings to the MS population at large, a subset analysis of tissue segmentation results was performed for those CIS cases with the top 10% of lesion loads. The mean (SD) lesion volume calculated using conventional BaMoS for this subset of cases was 14.1 ml (5.8 ml) at 1.5T and 15.5 ml (6.5 ml) at 3T. GM linear regression results between T1 and T2-FLAIR input to the new GIF database were β (SE) 1.029 (0.024) and R^2^ 0.997 for 1.5T and 1.022 (0.019), R^2^ 0.998 for 3T (Table 5). An example of GM segmentation performance in the case of high lesion load is presented in Fig. 9.

**Table 5:** Linear regression outputs for comparison of T1 and T2-FLAIR inputs into the new GIF database, for the 10% of cases with the highest lesion loads.

Change of sequence input-T1 vs T2-FLAIR input		β (SE)	R^2^
GM	1.5T	1.029 (0.024)	0.997
	3T	1.022 (0.019)	0.998
WM	1.5T	1.031 (0.035)	0.993
	3T	1.080 (0.024)	0.997
CSF	1.5T	0.905 (0.033)	0.992
	3T	0.821 (0.021)	0.996
TIV	1.5T	0.979 (0.012)	0.999
	3T	0.982 (0.015)	0.999

**Fig. 10:**
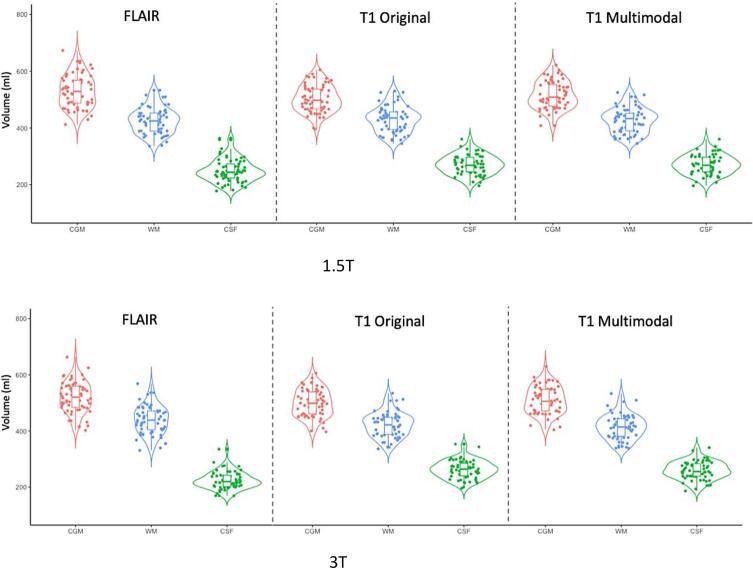
Violin plots displaying the actual volumes (in ml) returned per subject by tissue class and field strength – CSF, WM and GM – grouped by segmentation method. FLAIR = adapted GIF database with T2-FLAIR input; T1 original = standard GIF database with T1 input; T1 multimodal = adapted GIF database with T1 input. Violin plots were created using R.

The distribution of tissue segmentation volumes at the individual subject level in the T1 and T2-FLAIR groups are very similar, as demonstrated in violin plots by segmentation method for each of three tissue classes (CSF, WM and GM) and by field strength (Fig. 10).

Univariate analyses were computed for GM volume versus age for each segmentation method. GM volumes were significantly associated with TIV and age, which were therefore included as covariates for all subsequent models. Field strength was included as a fixed factor. Age was a significant covariate for all three of conventional T1 GIF (R^2^ = 0.999, standard error (SE) = 0.178, p = 0.001), T1 new GIF database (R^2^ = 0.999, SE = 0.182, p < 0.001), and T2-FLAIR new GIF database (R^2^ = 0.998, SE = 0.247, p = 0.005). Effect sizes for age, Cohen’s *f*, in each model were calculated for T1 GIF, effect size *f* = 0.36, and T2-FLAIR GIF, *f* = 0.27).

## 4 Discussion

In this study, we have investigated the performance of automated T2-FLAIR-only lesion and brain segmentation in a group of patients with CIS at different field strengths. It is common for clinical MS imaging protocols not to include a 3D-T1 sequence, limiting the use of conventional T1 or multi-sequence automated quantification techniques in clinical neuroradiology. We hypothesised that results of T2-FLAIR-only segmentation would provide comparable results to T1- and multi-sequence methods. Using a multi-centre population of CIS subjects, which benefitted from subjects having been scanned with 1.5T and 3T scanners in the same week, we compared the output of WM lesion and brain volume segmentation using conventional BaMoS and GIF algorithms with that from adapted T2-FLAIR-only versions. We showed that, with automated T2-FLAIR-only methods, lesion segmentation was comparable to conventional segmentation at 3T, and that at both 1.5T and 3T brain tissue segmentation was robust, with high R^2^ linear regression values and maintained discrimination of age-related brain volume change.

### 4.1 WM lesion segmentation

We used two sets of manual segmentations of white matter lesions in our CIS dataset to compare with automated results: 1. based on expert consensus reading of MS-specific lesions and 2. of all white matter hyperintensities, i.e. not specifically MS-identified lesions, at 1.5T and 3T. These varied quite considerably from each other, and automated segmentation reflected the latter manual scenario more closely. This indicates that automated segmentation algorithms can be limited in discriminating true MS lesions from any WMH. These other WMHs may include non-specific lesions more in keeping with vascular disease or normal aging, periventricular white matter bands and caps, or even image artefacts. They could also include true MS lesions, not captured by conservative criteria.

It is important to consider that this may be an inherent disadvantage in applying intensity-based methods of automated lesion segmentation to quantify MS-specific pathology. However, since we have also shown that total lesion volume difference between methods is small, as long as eventual end-users are aware of this limitation and apply it consistently as an adjunct to the radiologist’s visual assessment the discrepancy should not be impactful.

We demonstrated differences in lesion segmentation performance between field strengths, which we discuss further in section 4.3. At 1.5T, T2-FLAIR-only automated lesion segmentation was not significantly different from a manual segmentation method for all WM hyperintensities (manual method 2) and, at 3T, lesion volumes were comparable between conventional and T2-FLAIR-only segmentation. Proportional lesion volume differences were very small between the two automated methods at 3T. This contrasted with the situation at 1.5T, where lesion volumes were not comparable between the two automated methods and volume difference was higher.

As we were using a CIS subject population in this study, we expected WM lesion loads to be low, which made lesion segmentation method comparison challenging and produced dice scores which were relatively low. However, it is accepted that accurate automated lesion segmentation is easier where lesion load is higher (Carass et al., 2017). It will be important to expand on this study by applying our T2-FLAIR-only method to an MS population with higher lesion loads.

### 4.2 Brain tissue segmentation

We have shown that T2-FLAIR-only brain tissue segmentation provides similar results compared to the conventional T1 method, with very high R^2^ values and low standard error. Having used a no-intercept linear regression model for comparison, the coefficients quoted in Table 2, Table 3, Table 4 can be interpreted as straightforward multiplicative factors and their raw sizes demonstrate very minimal differences in brain tissue volume between change of GIF database, sequence input, and a combination of both changes. A subset analysis of cases with high lesion loads demonstrated maintained high tissue segmentation performance.

T2-FLAIR-only GIF segmentation was also effective in demonstrating biological effects in our study population, i.e. age remained a highly significant association using the T2-FLAIR-only method. Similar magnitude age-related effect sizes are seen when using a T1 input to the two different GIF databases as when changing between T1 and T2-FLAIR input to the new GIF database.

The encouraging results from this study point towards potential utility of T2-FLAIR-only automated brain tissue segmentation as a clinical tool for brain volume analysis, with further work needed to assess its validity in other MS phenotypes where more obvious parenchymal atrophy may be present. Currently the neurodegenerative aspect of MS is not routinely reported clinically, whilst being recognised as an important biomarker in the research setting that faces practical barriers for clinical adoption (Sastre-Garriga et al., 2020). Utilisation of automated segmentation tools could help to identify pathological brain atrophy in MS at the individual patient level (Sormani et al., 2017), but several technical barriers exist. A large proportion of clinical centres still use a 2D T2-FLAIR sequence in their protocols, and tools are available that measure central atrophy accurately from heterogeneous 2D T2-FLAIR data (Zivadinov et al., 2018). However centres are increasingly adopting a 3D sequence in line with most current guidance (Sastre-Garriga et al., 2020, Saslow et al., 2020, Filippi et al., 2019), making this work timely and relevant to the developing change in clinical practice. Beyond current clinical practice, these algorithms could be useful for integration of analysis of grey matter topology in patients with MS, such the construction of cortical networks (Collorone et al., 2019).

### 4.3 Field strength and acquisition

Our results show that T2-FLAIR-only tissue segmentation can be performed to a high level of robustness, with the knowledge that there are small multiplicative differences between T2-FLAIR-based and T1-based volumes. We have also shown that there are variations in performance between the field strengths, with different multiplicative factors and in general slightly lower variance at 3T than 1.5T, as seen in Table 4. Likewise for lesion volumetry, where we saw that lesion volumes were overestimated at 1.5T, this should be considered when using automated segmentation tools in clinical practice; results for different patients and at different timepoints may not be directly comparable if not consistently scanned at the same field strength (Han et al., 2006, Lysandropoulos et al., 2016).

Within a single field strength, differences in scanners and image acquisition parameters – which is a fundamental issue in the clinical setting - can impact on the performance of automated segmentation algorithms (Biberacher et al., 2016). At present there is limited experience in standardising T2-FLAIR acquisition protocols, in contrast to the advances that have been seen with T1 imaging (George et al., 2019, Jack et al., 2015). In the case of T1 imaging, automated segmentation methods have been shown to be sensitive to differences in sequence parameters contributing to volumetric errors of up to 4–5% at 1.5T on the same scanner, which would obscure biological effects (Haller et al., 2016). Efforts have been led by the Alzheimer’s Disease Neuroimaging Initiative (ADNI) to standardise protocols and remove these sources of bias (Brewer, 2009). Work towards adoption and harmonisation of 3D T2-FLAIR acquisition, at least across a single clinical service, and ultimately across centres to facilitate research and reference data sharing, may address a significant amount of the variability. MS-applicable T2-FLAIR harmonisation initiatives are being made in earnest by groups like MAGNIMS, NAIMS and CMSC (Saslow et al., 2020). Their adoption would greatly facilitate the validation and interpretation of automated segmentation algorithm outputs in the clinical setting.

### 4.4 Limitations

There were some limitations to this study. Whilst the dataset we used was multi-centre and multi-vendor, providing a good mimic of a clinical dataset, numbers of subjects from each centre were not balanced and image homogeneity was not guaranteed. However, this does mean that the results are likely to be more generalisable. Since we used a CIS cohort, we were not able to address the effect of disease-mediated brain atrophy on T2-FLAIR-only brain tissue segmentation. Whilst we did not include data from other MS phenotypes, a subset analysis of CIS cases with high lesion loads showed consistent segmentation performance. Further testing of T2-FLAIR GIF with other MS phenotypes is needed to establish its clinical utility across the disease spectrum. Additionally, we were not able to assess scan-rescan reproducibility within each field strength for brain segmentation measurements.

### 4.5 Conclusions

We have shown that T2-FLAIR-only automated segmentation of brain volumes can be reproducible and comparable to conventional T1 or dual-modality methods, although with lower lesion segmentation robustness at lower field strengths. Further validation with other MS phenotypes, as well as work towards clinical image acquisition harmonisation, can further improve clinical validation and integration of T2-FLAIR-only WM lesion volume and brain atrophy analysis for radiological MS reporting.

## Declaration of Competing Interest

The authors declare that they have no known competing financial interests or personal relationships that could have appeared to influence the work reported in this paper.
