# Supplementary material for: FLAIR-only joint volumetric analysis of brain lesions and atrophy in clinically isolated syndrome (CIS) suggestive of multiple sclerosis
Source: Neuroimage Clin. 2020 Dec 25;29:102542. doi: 10.1016/j.nicl.2020.102542 (PMC7804983; doi:10.1016/j.nicl.2020.102542)
Supplement: Supplementary Data 1 [file mmc1.docx]

# 6. Supplementary material

Table 6. MRI sequence parameters by centre, for 1.5T and 3T.

| *MAGNIMS Centre* | *1.5T* | | | | *3T* | | | |
| --- | --- | --- | --- | --- | --- | --- | --- | --- |
|  | Vendor | Parameter | 3D T1 | 3D FLAIR | Vendor | Parameter | 3D T1 | 3D FLAIR |
| VU University Medical Center Amsterdam | GE Signa HDxt | Type | GRE | TSE | GE Discovery MR750 | Type | GRE | TSE |
|  |  | Slice orientation | Sag | Sag |  | Slice orientation | Sag | Sag |
|  |  | Measured voxel size (mm) | 1.0x1.0x1.0 | 1.4x1.4x1.2 |  | Measured voxel size (mm) | 1.0x1.0x1.0 | 1.1x1.1x1.2 |
|  |  | TR (ms) | 12.4 | 6500 |  | TR (ms) | 8.2 | 8000 |
|  |  | TE (ms) | 5.2 | 115 |  | TE (ms) | 3.2 | 130 |
|  |  | Flip angle (degrees) | 12 |  |  | Flip angle (degrees) | 12 |  |
|  |  | Turbo factor |  | 191 |  | Turbo factor |  | 230 |
|  |  | Inversion times (ms) | 450 | 1994 |  | Inversion times (ms) | 450 | 2340 |
| University Hospital Basel | Siemens Avanto | Type | GRE | TSE | Siemens Verio | Type | GRE | TSE |
|  |  | Slice orientation | Sag | Sag |  | Slice orientation | Sag | Sag |
|  |  | Measured voxel size (mm) | 1.0x1.0x1.0 | 1.0x1.0x1.0 |  | Measured voxel size (mm) | 1.0x1.0x1.0 | 1.0x1.0x1.0 |
|  |  | TR (ms) | 2700 | 6000 |  | TR (ms) | 1570 | 5000 |
|  |  | TE (ms) | 3.37 | 352 |  | TE (ms) | 2.67 | 402 |
|  |  | Flip angle (degrees) | 8 |  |  | Flip angle (degrees) | 9 |  |
|  |  | Turbo factor |  | 141 |  | Turbo factor |  | 141 |
|  |  | Inversion times (ms) | 950 | 2200 |  | Inversion times (ms) | 900 | 1800 |
| St. Josef Hospital Bochum | Siemens Avanto | Type | GRE | TSE | Philips Achieva | Type | GRE | TSE |
|  |  | Slice orientation | Sag | Sag |  | Slice orientation | Sag | Sag |
|  |  | Measured voxel size (mm) | 1.1x1.1x1.0 | 1.0x1.0x1.0 |  | Measured voxel size (mm) | 1.1x1.1x1.0 | 1.0x1.3x1.0 |
|  |  | TR (ms) | 10 | 4800 |  | TR (ms) | 10 | 5000 |
|  |  | TE (ms) | 4.6 | 291 |  | TE (ms) | 4.2 | 354 |
|  |  | Flip angle (degrees) | 8 |  |  | Flip angle (degrees) | 15 |  |
|  |  | Turbo factor |  | 204 |  | Turbo factor |  | 182 |
|  |  | Inversion times (ms) | 1000 | 1650 |  | Inversion times (ms) | 1100 | 1900 |
| UCL Institute of Neurology London | Siemens Avanto | Type | GRE | TSE | Philips Achieva | Type | GRE | TSE |
|  |  | Slice orientation | Sag | Sag |  | Slice orientation | Sag | Sag |
|  |  | Measured voxel size (mm) | 1.0x1.0x1.0 | 1.0x1.0x1.0 |  | Measured voxel size (mm) | 1.0x1.0x1.0 | 1.2x1.2x1.2 |
|  |  | TR (ms) | 1900 | 6500 |  | TR (ms) | 6.9 | 8000 |
|  |  | TE (ms) | 3.37 | 202 |  | TE (ms) | 3.1 | 388 |
|  |  | Flip angle (degrees) | 15 |  |  | Flip angle (degrees) | 8 |  |
|  |  | Turbo factor |  | 125 |  | Turbo factor |  | 120 |
|  |  | Inversion times (ms) | 1100 | 2000 |  | Inversion times (ms) | 821 | 2400 |
| Hospital Clínico San Carlos Madrid | GE Signa HDxt | Type | GRE | TSE |  | Type | GRE | TSE |
|  |  | Slice orientation | Sag | Sag |  | Slice orientation | Sag | Sag |
|  |  | Measured voxel size (mm) | 0.98x0.98x1.0 | 0.98x0.98x1.0 |  | Measured voxel size (mm) | 0.98x0.98x1.0 | 0.98x0.98x1.0 |
|  |  | TR (ms) | 10 | 6000 |  | TR (ms) | 10 | 6000 |
|  |  | TE (ms) | 4.2 | 136 |  | TE (ms) | 4.2 | 135 |
|  |  | Flip angle (degrees) | 12 |  |  | Flip angle (degrees) | 12 |  |
|  |  | Turbo factor |  | 220 |  | Turbo factor |  | 220 |
|  |  | Inversion times (ms) | 450 | 1837 |  | Inversion times (ms) | 450 | 1840 |
| Sapienza University of Rome | Siemens Avanto | Type | GRE | TSE | Siemens Verio | Type | GRE | TSE |
|  |  | Slice orientation | Sag | Sag |  | Slice orientation | Sag | Sag |
|  |  | Measured voxel size (mm) | 1.0x1.0x1.0 | 1.2x1.2x1.3 |  | Measured voxel size (mm) | 1.0x1.0x1.0 | 1.0x1.0x1.0 |
|  |  | TR (ms) | 1900 | 6500 |  | TR (ms) | 1900 | 5000 |
|  |  | TE (ms) | 3.37 | 202 |  | TE (ms) | 2.93 | 395 |
|  |  | Flip angle (degrees) | 15 |  |  | Flip angle (degrees) | 9 |  |
|  |  | Turbo factor |  | 125 |  | Turbo factor |  | 141 |
|  |  | Inversion times (ms) | 1100 | 2000 |  | Inversion times (ms) | 900 | 1800 |

*GE=General Electric; Sag=sagittal; GRE=gradient echo; TSE= turbo spin-echo; TE=echo time; TR=repetition time*

Table 7 Akaike Information Criterion (AIC) calculations for model fit for both intercept and no-intercept models, for cortical GM volume comparison between GIF methods.

| **GM volume**  **comparison** | **Field strength** | **AIC** | |
| --- | --- | --- | --- |
|  |  | **Intercept** | **No intercept** |
| T1 (original vs. new database) | 1.5 | 234.63 | 233.55 |
|  | 3 | 220.83 | 221.78 |
| T1 vs T2-FLAIR (new database) | 1.5 | 432.00 | 430.00 |
|  | 3 | 397.06 | 395.23 |

Table 8 Linear regression outputs for comparison of T1 inputs into the original T1-only and new GIF database using a no-intercept model.

| **Change of GIF database and sequence**  **-**  **T1 vs**  **T2-FLAIR input** | | **Coefficient** | **R²** | **Standard error** |
| --- | --- | --- | --- | --- |
| GM | **1.5T** | 1.054 | 0.996 | 0.008 |
|  | **3T** | 1.041 | 0.998 | 0.006 |
| WM | **1.5T** | 0.987 | 0.996 | 0.008 |
|  | **3T** | 1.036 | 0.995 | 0.009 |
| CSF | **1.5T** | 0.913 | 0.988 | 0.012 |
|  | **3T** | 0.850 | 0.994 | 0.008 |
| TIV | **1.5T** | 0.984 | 0.999 | 0.004 |
|  | **3T** | 0.988 | 0.999 | 0.004 |

Table 9 Linear regression outputs for comparison of conventional T1 GIF and T2-FLAIR GIF – i.e. change of both GIF database and sequence input – using a non-intercept model.

| **Change of GIF database**  -  **T1 input** | | **Coefficient** | **R²** | **Standard error** |
| --- | --- | --- | --- | --- |
| GM | **1.5T** | 0.979 | 0.999892 | 0.001 |
|  | **3T** | 0.981 | 0.999886 | 0.001 |
| WM | **1.5T** | 1.004 | 0.999686 | 0.002 |
|  | **3T** | 1.014 | 0.999512 | 0.003 |
| CSF | **1.5T** | 1.034 | 0.999566 | 0.003 |
|  | **3T** | 1.013 | 0.999445 | 0.003 |
| TIV | **1.5T** | 0.988 | 0.999923 | 0.001 |
|  | **3T** | 0.988 | 0.999926 | 0.001 |


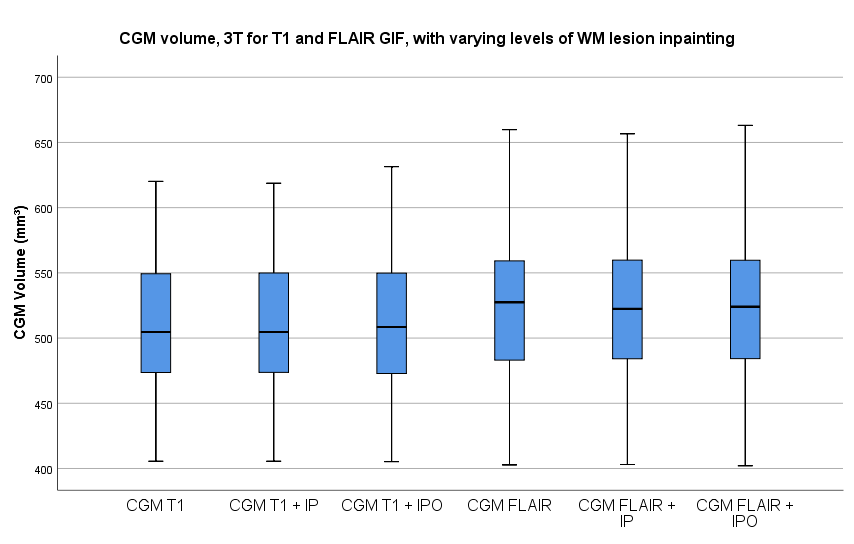


GM volume (ml)

Figure 11 Boxplots for cortical GM volume by method at 1.5T (T1 or T2-FLAIR input into the new GIF database), by varying levels of WM lesion inpainting. IP = inpainting with manual WM lesion segmentation; IPO = inpainting with BaMoS outliers.


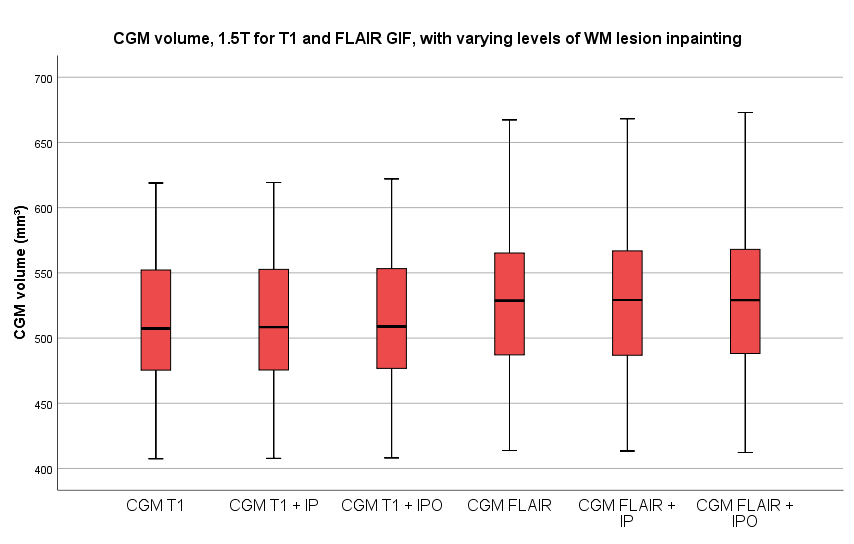


GM volume (ml)

**Figure 12 Boxplots for cortical GM volume by method at 3T (T1 or T2-FLAIR input into the new GIF database), by varying levels of WM lesion inpainting. IP = inpainting with manual WM lesion segmentation; IPO = inpainting with BaMoS outliers.**

GM volume (ml)

Table 10 Mean cortical GM volume as a percentage of TIV, by GIF segmentation method, and by WM lesion inpainting method, for 1.5T.

| CGM segmentation method, 1.5T | WM lesion inpainting method | Mean GM volume as % of TIV | SD |
| --- | --- | --- | --- |
| T1 (new GIF database) | None | 31.64 | 1.15 |
|  | Manual | 31.67 | 1.15 |
|  | BaMoS | 31.81 | 1.22 |
| T2-FLAIR (new GIF database) | None | 33.20 | 1.62 |
|  | Manual | 33.22 | 1.63 |
|  | BaMoS | 33.29 | 1.66 |

Table 11 Mean cortical GM volume as a percentage of TIV, by GIF segmentation method, and by WM lesion inpainting method, for 3T.

| CGM segmentation method, 3T | WM lesion inpainting method | Mean GM volume as % of TIV | SD |
| --- | --- | --- | --- |
| T1 (new GIF database) | None | 31.80 | 1.10 |
|  | Manual | 31.81 | 1.10 |
|  | BaMoS | 31.89 | 1.13 |
| T2-FLAIR (new GIF database) | None | 33.23 | 1.43 |
|  | Manual | 33.15 | 1.31 |
|  | BaMoS | 33.24 | 1.43 |
